# The relation of forward head posture with back muscle endurance in primary school children: a cross-sectional study

**DOI:** 10.1186/s43161-022-00105-8

**Published:** 2022-11-23

**Authors:** Asmaa Hosny Abd-Elshafy, Gehan Hassan El-Meniawy, Wael S. Abu El Azm, Mahmoud S. El Fakharany

**Affiliations:** 1 Department of Physical Therapy for Pediatrics, Faculty of Physical Therapy, Cairo University, Cairo, Egypt; 2 Dean of Faculty of Physical Therapy, Cairo University, Cairo, Egypt; 3 Department of Statistics, Faculty of Commerce, Zagazig University, Zagazig, Egypt

**Keywords:** Forward head, Muscle endurance, Craniovertebral angle, School children

## Abstract

**Background:**

The goal of this study was to look at the correlation between forwarding head posture and back muscle endurance in 288 primary school healthy active children aged 7–10 years old in public schools in Sheblanga, Benha, Qalubyia governorate (170 boys and 118 girls).

**Methods:**

A cross-sectional study was conducted on elementary school children to see whether there was a link between forwarding head posture and back muscle endurance. The photogrammetry technique of craniovertebral angle (CVA) was employed to quantify forward head position, and the Biering Sorensen test was utilized to evaluate isometric back muscle endurance.

**Results:**

The distribution of head posture in the study group revealed 132 (46%) children with advancing head posture and 156 (54%) children with normal posture. In the study group, the connection between CVA and trunk extensor endurance was moderately significant (*r* = 0.4, *p* = 0.0001). The trunk extensor endurance of children with advancing heads was significantly lower than that of children with normal postures (*p* = 0.0001).

**Conclusions:**

There is a link between forwarding head posture (FHP) and back muscle endurance.

## Background

Long-term use of digital media, especially smartphones, frequently results in negative changes in head and neck posture, rounding the shoulders and pulling the head forward [1]. The anterior orientation of the cervical spine is referred to as forwarding head posture (FHP) [2] and is linked to muscular imbalance, discomfort, fatigue, and limited cervical spine motion [3].

By increasing external flexion torque and putting a greater burden on the extensors and some connective tissues, a forward head posture (FHP) produces various negative symptoms such as neck ache, shoulder pain, upper back pain, persistent headaches, increased curvature of the spine, and scapular dyskinesia [4]. In the long run, this incorrect posture can injure not only the cervical vertebrae and ligaments but also the structures around the lumbar area [5]. Muscular endurance refers to a muscle’s ability to exert a force repeatedly over time. It also refers to a muscle’s ability to sustain a fixed or static contraction, i.e., the ability to apply force and keep it there [6].

Epidemiological research found that spinal postural deviations predominated in children and adolescents, with forward head posture (FHP) and protracted shoulder posture (PS) being two of the most prevalent postural deviations [7]. According to Harrison (1999), “bad posture of any section of the spinal column will create abnormal stresses in the entire cord and neurological system, whereas appropriate posture will reduce these stresses.” Theoretically, cervical spine positions can be believed to have direct impacts on the thoracic cord, lumbar cord, and lumbosacral nerve roots [8].

Aries et al. [9] discovered a positive link between neck flexion and neck pain, implying an increased risk of neck pain for people who study with their necks at least 20° flexed for more than 70% of the time. Furthermore, when using cell phones, all of our participants exhibited significant neck flexion. “Text neck,” a twenty-first century syndrome, is a name developed from the onset of cervical spine degeneration caused by the repeated stress of frequent forward head flexion when staring down at mobile device screens and “texting” for extended periods [10]. The effects of forwarding neck flexion extend beyond discomfort to lead to further problems.

As the eyes are forced to focus on a nearby object, the effects of extended neck flexion can often contribute to nearsightedness, eye strain, or dry eyes [11]. A new study reveals a link between forward-leaning postures used while texting, studying, surfing the web, emailing, and playing video games and hyperkyphosis, which is linked to pulmonary disease and cardiovascular difficulties [12]. It was anticipated that forwarding head posture (FHP) and back muscle endurance in elementary school children were related. As a result, our research focuses on the relationship between abnormal cervical spine posture and back muscle endurance.

## Methods

### Participants

#### Sample size

The number of students from grade 2 to grade 5 in local schools was 1147. The estimated prevalence of forwarding head posture in primary school children was 53% [13]. With an expected prevalence of 53% and precision of 2% with a 95% confidence interval, the required sample size was 288 according to the Daniel formula:

$$\mathrm n=\mathrm{NZ}^2\;\mathrm P(1-\mathrm P)/\;\mathrm d^2\;(\mathrm N-1)\;+\mathrm Z^2\;\mathrm P(1-\mathrm P)\;$$

where *n* is the sample size, *N* indicates the population size, *Z* resembles the statistic corresponding to the level of confidence, *P* reflects the expected prevalence, and *d* shows the precision level [14].

288 primary school healthy active children in public schools at Sheblanga, Benha, Qalubyia governorate (170 boys and 118 girls) aged 7–10 years; their average value is ± SD; age, weight, and height were 8.94 ± 1.02 years, 31.86 ± 6.63 kg, and 131.5 ± 8.79 cm D). Those children have taken part in this cross-sectional study using convenient sampling. The examiner contacted the participants through the school administration. The inclusion criteria were having a craniovertebral angle (CVA) less than 50°, their age ranging from 7 to 10 years, and being from both genders. The exclusion criteria were as follows: subjects with pronated feet or leg length discrepancy, history of trunk or neck surgery, participation in any sports activities, and subjects with a neurological disorder (upper motor neuron lesion and lower motor neuron lesion).

When the parents of the children were questioned if their children had any vision, hearing, or dental issues, they affirmed that their children did not. Ethical committee approvals from Cairo University’s Faculty of Physical Therapy, as well as written agreement from children’s parents, were obtained after complete information about the nature of the examination, objectives, and benefits of the study was provided. From 2019 to 2021, this study was carried out in gymnastic laboratories at Sheblanga primary schools during the children’s free time.

### Procedures

The photogrammetry approach was used to assess forward head posture (FHP) by measuring craniovertebral angle (CVA) in the standing position. Children with CVA levels less than 49° were thought to have FHP [15]. The Biering-Sorenson test was used to measure the static endurance of trunk muscle extensors. Endurance scores were measured in seconds, and the test was terminated if the subject could not maintain the tests’ defined position based on special considerations for each test or experienced discomfort or pain [16].

To avoid discomfort and pain among participants, the endurance test and CVA measurement procedures were performed just once after all members were given point-by-point clarifications of the methodologies. Following the collection of all information, the inspector performed test procedures to ensure the exact appliance. Participants were also given verbal and tactile input to help them maintain their endurance test positions properly.

**Fig. 1:**
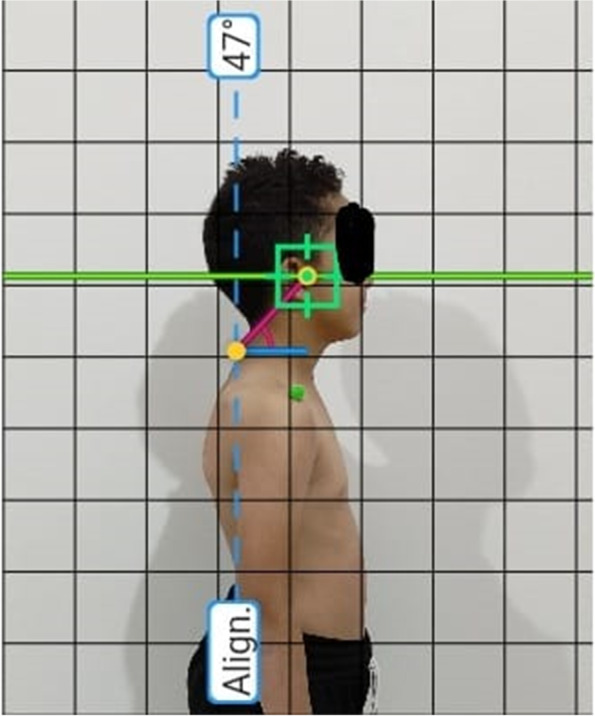
Measuring CVA (colorful styrofoam balls were placed on C7 and the tragus of the ear)

Photographs were shot from the lateral left and right perspectives. The digital camera used for image capture was set up on a 1 m tripod 1.5 m away from the subjects in a standing position on a fixed base with no tilt or rotation. Figure 1 shows how colorful styrofoam balls were inserted in specified anatomical landmarks (C7 and the tragus of the ear) using double-faced adhesive tape.

The CVA depicts the angle formed between the spinous process of the seventh neck vertebrae (C7) and the ear tragus with a horizontal line passing through the seventh neck vertebrae. After being obtained, the photographic images were transferred to a computer for the assessment using the postural evaluation computer application (Postural Assessment Software PAS). Picture calibration, anatomical landmark recognition, and computation of the comparing body’s angles and distances are all part of information handling.

A cross-format screen design screen cursor was used to encourage the recognized center of ball-shaped landmarks, and the evaluator will be allowed to use the software’s zoom capability. Angles and distances on the body were computed in degrees and centimeters, respectively. PAS is a precise programming for measuring points and distances with high inter and intra-rater reliabilities, and it should be regarded as a useful and dependable instrument for posture assessment [17].

**Fig. 2:**
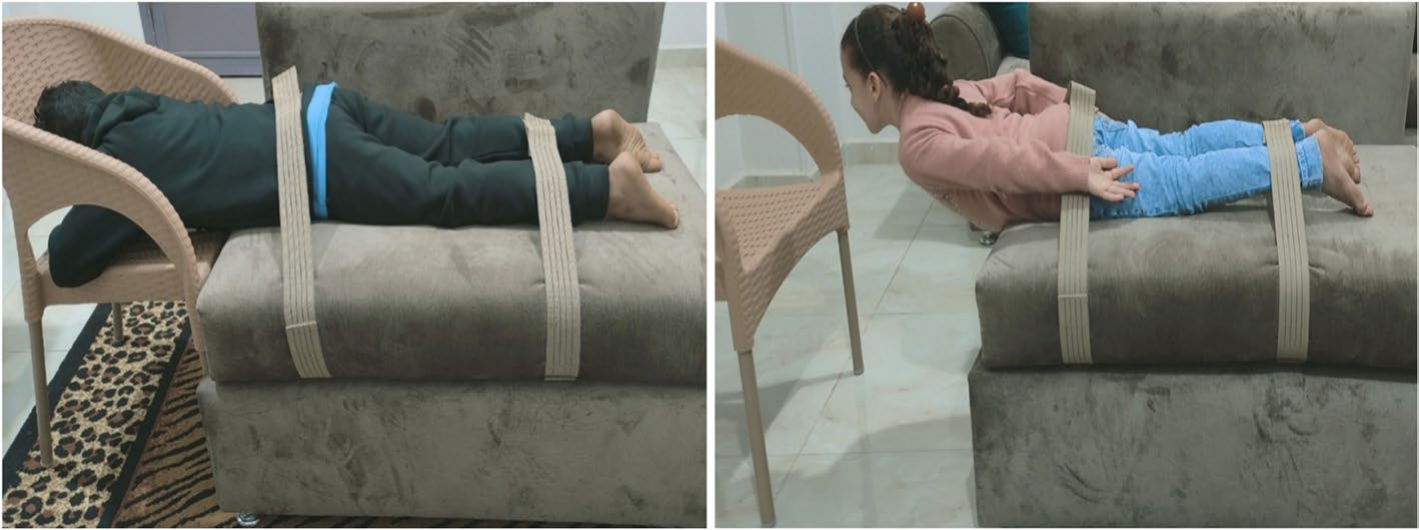
Beiring Sorensen Test

The Biering-Sorenson test is the most often used method for assessing the isometric endurance of trunk extensor muscles [18]. The subject was requested to lie prone, with the lower body secured to the comfortable table by two sturdy straps at the hips and ankles. The upper body was extended over a stool, away from the table. The individual was instructed to release the table while holding the upper extremities and chest in extension. It was told to lift the upper body off the floor and maintain a horizontal position for as long as feasible [16]. The examiner calculated the endurance time using a stopwatch as long as the position was maintained, as indicated in Fig. 2 [19].

### Subjects characteristics

Two hundred eighty-eight primary school children (118 girls and 170 boys) took part in the current study. Their average value ± SD age, both weight and height were 8.94 ± 1.02 years, 31.86 ± 6.63 kg, and 131.5 ± 8.79. Table 1 showed the subject characteristics.

**Table 1:** Descriptive statistics for age, weight, and height of the study group

	Mean ± SD	Maximum	Minimum
**Age (years)**	8.94 ± 1.02	10	7
**Weight (kg)**	31.86 ± 6.63	54	20
**Height (cm)**	131.5 ± 8.79	153	100

*SD* standard deviation

### CVA and trunk extensor endurance of the study group

The mean ± SD CVA of the study group was 50.17 ± 3.97°. According to the study group’s head posture distribution, there were 132 (46%) children with forwarding head posture and 156 (54%) children with normal posture, as indicated in Table 2.

**Table 2:** The frequency distribution of head posture in the study group

	Head posture distribution	
	Forward head posture	Normal posture
**No. (%)**	132 (46%)	156 (54%)
**Total**	288 (100%)	

The mean ± SD trunk extensor endurance of the study group was 99.75 ± 52.1 sec (Table 3).

**Table 3:** Descriptive statistics of the CVA and trunk extensor endurance of the study group

	Mean ± SD	Minimum	Maximum
**CVA (degrees)**	50.17 ± 3.97	40	63.5
**Trunk extensor endurance (s)**	99.75 ± 52.1	31	301

*SD* Standard deviation

### Relationship between CVA and trunk extensor endurance

**Fig. 3:**
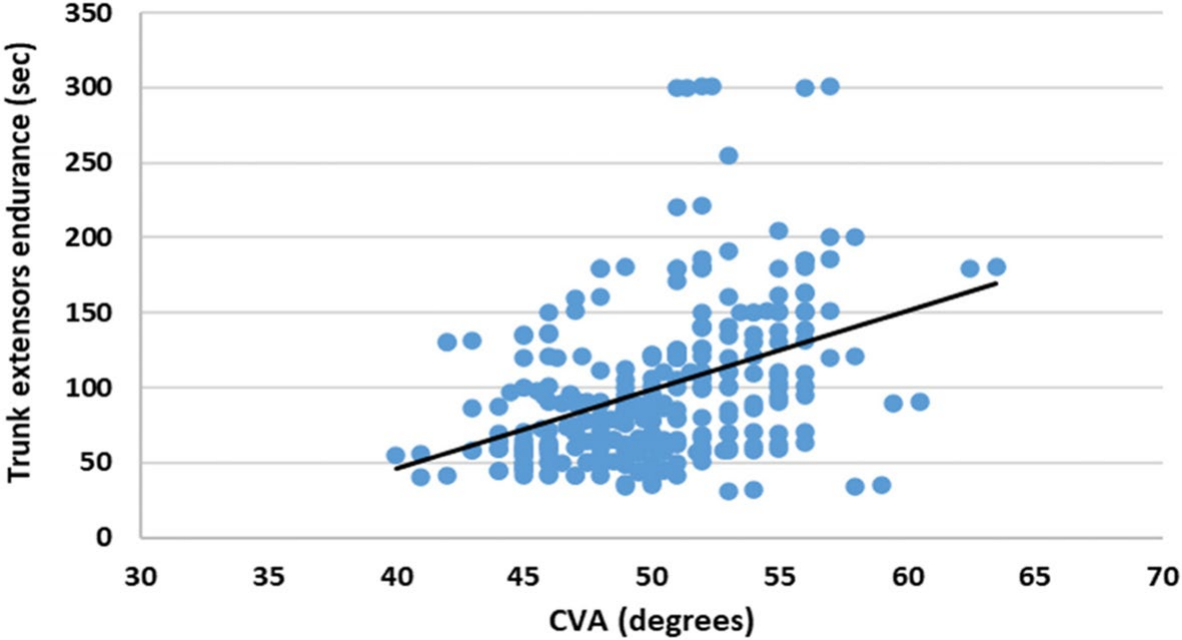
Correlation between CVA and trunk extensor endurance in the study group

The correlation between CVA and trunk extensor endurance in the study group was positive, prominent relation (*r* = 0.4, *p* = 0.001) (Fig. 3).

### Comparison of trunk extensor endurance between children with forward head posture (FHP) and children with normal posture

There was a significant decrease in trunk extensor endurance of children with forwarding heads compared with that of children with normal posture (*p* = 0.0001) (Table 4).

**Table 4:** Comparison of trunk extensor endurance between children with forward head posture (FHP) and children with normal posture

	Children with forward head posture (***N*** = 132)	Children with normal posture (***N*** = 156)	MD	***t*** value	***p*** value
	Mean ± SD	Mean ± SD			
**Trunk extensors endurance (s)**	78.13 ± 32.15	118.04 ± 58.47	−39.91	−6.99	0.0001

*SD* Standard deviation, *MD* Average disparity, *p* value, the probability value

## Results

### Statistical analysis

To present the individuals’ demographic and measurable data, descriptive statistics in the form of the mean, standard deviation, minimum, maximum, and frequency were used. The Shapiro–Wilk test was used to ensure that the data was regularly distributed. The Pearson correlation coefficient was used to assess the relationship between CVA and trunk extensor endurance.

Interpretation guidelines for the correlation 0 mean that there is no linear relationship. A weak positive (negative) linear relationship is indicated by values between 0 and 0.3 (0 and 0.3). A substantial positive (negative) linear connection is indicated by values between 0.3 and 0.7 (−0.3 and −0.7). A significant positive (negative) linear relationship is indicated by values between 0.7 and 1.0 (−0.7 and −1.0) (Ratner, 2009) [20].

CVA and trunk extensor endurance were compared between children with forward head posture and those with normal posture using an unpaired *t* value. For all statistical tests, the significance level was set at *p* 0.05. The statistical package for social studies (SPSS) version 25 for Windows was used to run all statistical analyses.

## Discussion

The major objective is to figure out if there was a link between forwarding head position (FHP) and back muscle endurance in school children. The distribution of head posture in the study group revealed that 132 (46%) of the children had forward head posture and 156 (54%) had normal posture. In our study group, there was a moderately positive connection between CVA and trunk extensor endurance. When compared to children with normal posture, trunk extensor endurance was significantly lower in children with forwarding heads.

Fascia connects the neck muscle to the trunk muscle, according to Tomas and Myers (2014) [21]. As a result, if neck problems need to be corrected, trunk posture should be taken into account. Furthermore, Diab (2012) discovered that FHP corrective exercises improved spinal posture in patients with lumbosacral radiculopathy or young adult idiopathic scoliosis [22]. Drozda and Lewandowski (2011) backed up the findings of this study, stating that poor posture in school children leads to degenerative changes in the spine, functional disorders, poor motor skill performance, and, ultimately, lower quality of life [23].

According to Hlavenka et al. (2017) [24], hyperextension of the upper cervical spine found in FHP may contribute to lower trunk and back muscle endurance and, in the long run, may predispose people to back injury in the future when performing high-demand tasks like lifting. The findings of this study corroborate those of Myers (2010), who stated that forward head posture causes eccentric contraction of the posterior cervical muscles, which influences balance control by delivering tension to plantarflexion of the ankle via the fascia’s effect, and who also summarized fascia’s connecting human body muscles into 11 major myofascial meridians.

The superficial backline is one of these meridians. It connects the gastrocnemius to the hamstrings, which are connected to the ischial tuberosity, sacrotuberous ligament, thoracolumbar fascia, erector spinae, iliocostalis, epicranial, galea aponeurotica, and frontalis muscles, to transmit tension from the head or gastrocnemius to other connected segments. Forward head posture may impact balance due to the continual eccentric contraction of extension muscles in the neck, which is communicated to the gastrocnemius via the myofascial meridian [25].

Long-term bad posture can result in muscle grinding and muscular imbalances, as well as decreased muscle efficacy and increased mechanical stress, all of which can result in discomfort [26]. According to Black et al. (1996), a change in lumbar posture was connected to a compensatory adjustment in cervical position [27]. Repetitive static and dynamic stress of the spine, according to Van et al. (2003), is a risk factor for low back, shoulder, and neck discomfort in both adults and children [28].

Maintaining the head forward for lengthy periods, according to Szeto et al. [29] and Moore [30], can result in musculoskeletal diseases such as “upper crossed syndrome,” which combines reduced lordosis of the lower cervical vertebrae combined with kyphosis of the upper thoracic vertebrae.

### Limitations

Coronavirus is a virus that causes the death of people. For the children’s safety during the initial wave of coronavirus, several schools refused us access despite our security clearance, and the state canceled school education for a long time due to coronavirus.

## Conclusions

Forward head position is a common mechanical issue among school children, and it is related to trunk muscle endurance. Forward head posture should be corrected to avoid future difficulties with the spine or posture.

## Data Availability

The data that support the findings of this study are available from the corresponding author, MS, upon reasonable request.

## Abbreviations

FHP: Forward head posture
PS: Protracted shoulder
CVA: Craniovertebral angle
PAS: Postural Assessment Software
